# Fucus Marina

**Published:** 1885-08

**Authors:** B. Roemer


### Fucus Marina.

By B. Roemer, M.D.

Few genus in the vegetable kingdom present as great a variety of usefulness as the various kinds of fuci, algæ and coniferæ, belonging to almost the lowest types of vegetable organization, to the Cryptogamia Algæ, sexually considered, and to the natural order of Algaceæ, the former the genus Cryptogamia and the latter the order Algæ. It is well, however, to remark here that different botanists have classed some species under other divisions, since the Chondrus crispus, Carrageen or Irish Moss, is a Fucus by Linnæus, a Chondrus by Greville, and a Sphærococcus by Agarrh.

For centuries, the sea-weeds, seatang (Germ. Seetang), have been made use of as powerful manures, their fertilizing effects extending over more than one season. In the north of Scotland and Orkney Islands, the *fucus digitatus* is preferred on account of its greater strength. The most of them yield, on dilution with water, a large proportion of mucilage and by distillation water, but no ammonia. The residuum contains sea-salt, iodine, bromine, etc., and the carbonates of soda and potash.

The medical profession has for centuries employed these Cryptogams in the treatment of various diseases.

Perhaps, the oldest use made of fucus was in form of a poultice to swellings, bruises, etc. Ancient sages of Scandinavia and Denmark speak of soothsayers crushing sea-weed between stones and applying the moistened pulp to the affected parts. The *fucus vesiculosus* ranks, perhaps, next in order, being burnt in the open air, its black powder was administered internally, like spongia usta, in the treatment of glandular enlargements. Spongia, now admitted to be a polymorphous animal, as spongia usta, resembles in its chemical nature the fucus, and was at one time highly lauded in goitre, glandular swellings generally, scrofula and cutaneous eruptions. After having been much derided as to its efficacy in these diseases, its virtues were admitted after the discovery of iodine, and the early use is almost an instinctive medication. The black powder of burnt fucus vesiculosus was known under the name Æthiops vegetabilis. Its juice was also administered internally with its local application for the same diseases.

But this article was especially undertaken to draw the attention of the medical profession to a species called *Fucus Marina*, of which the Peacock Chemical Company, of St. Louis, is preparing an elixir. Having had at my disposal about four pounds of this preparation, which I divided among eight patients, I am prepared to give a favorable opinion of its therapeutic value as claimed. Not intended to replace any of the usually employed antiperiodics, especially quinine, it owes its virtue rather to an alterative quality, and having prescribed it in six cases of pronounced malaria, I find the result as follows :

All these cases had been under a quinine treatment. Whenever a hepatic complication was diagnosed, I gave:

| | | |
|---|---|---|
| ℞ | Mass. pil. Hydrarg. - - - | 3 grs. |
| | Quin. Sulph. - - - | 4 grs. |
| | Ol. Piper. Nigr. - - - | 1 prop. |
| | Ex. Hyosciam. Nigr. - - | ½ gr. |
| M. | Fiat. Pil. 2. | |

To be taken at night and followed next morning, if required, by a mild aperient. During the day I usually order 10 to 15 grains of quinine, and repeated on the next. The quasi typhoid character of the malaria remained, however, unchanged, and heretofore I altered my treatment to different remedies, omitting the quinine. The elixir of fucus marina, however, in the number of cases specified, gave me perfect satisfaction; in every instance, cutting the febrile exacerbation short and hastening convalescence. This result was especially marked in four cases, which had had a preliminary quinine treatment; in the two remaining cases, while recovery was perfect, three or four days additional were required, the quinine treatment being isochronic with the exhibition of the elixir. Thus, in any event, the course of malaria is materially checked by Fucus Marina, and, the system (secretions, etc.) having been properly prepared, it is my opinion that the elixir alone will give the best results.

Believing that the prominent therapeutic character of Fucus Marina is alterative, I prescribed the remedy in two cases of cachexia, scrofulous and syphilitic. The result, thus far, is gratifying, but time is required for a proper report. I will state, however, that in a mixed treatment for the disease I omitted smilax off, and stillingia as a vehicle altogether, and gave instead full doses of Fucus Marina, repeated four to six times a day, continuing the inunction of mercurials. A success in these cases would do away with the annoying complications resulting from large doses of the iodide of potash.

P. S.—Since writing the above short synopsis on the therapeutic value of Fucus Marina, several weeks have elapsed, and I can now add that the case of *secondary syphilis* is progressing very favorably under its use, it, the *Fucus Marina*, as prepared by the *Peacock Chemical Co.*, of St. Louis, fully and thoroughly taking the place of Iodide of Potassium in the mixed treatment, with this very desirable advantage over the salt of Iodine and Potash, that the stomach and digestive powers are not only left intact, but are really invigorated. Additional cases of *Malaria* have received, as already reported, prompt and lasting relief.

